# Supplementary material for: R2R - software to speed the depiction of aesthetic consensus RNA secondary structures
Source: BMC Bioinformatics. 2011 Jan 4;12:3. doi: 10.1186/1471-2105-12-3 (PMC3023696; doi:10.1186/1471-2105-12-3)

SAM-chlorobi

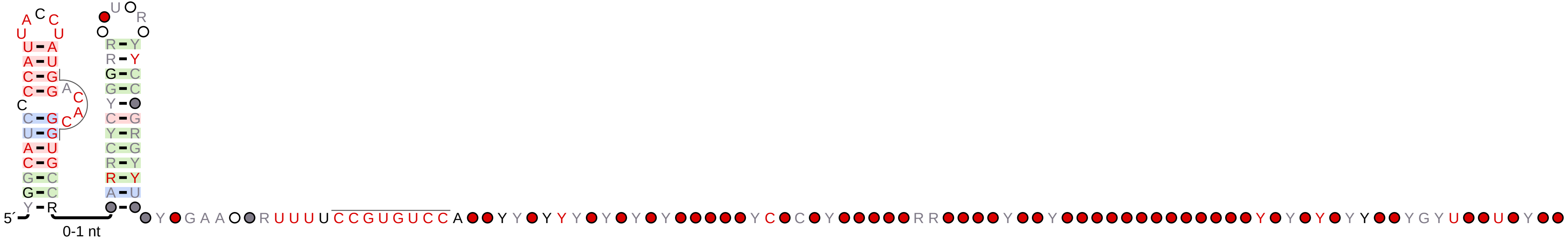

SAM-chlorobi NC_002932.3/706042-705555:S

Shrinking nucs & bonds using
nucShrinkWithCircleNuc and pairBondScaleWithOneSeq
because circle_nuc or #=GR ... CLEAVAGE was used
Set these vars to 1 using SetDrawingParam
to disable. See note1 in manual.

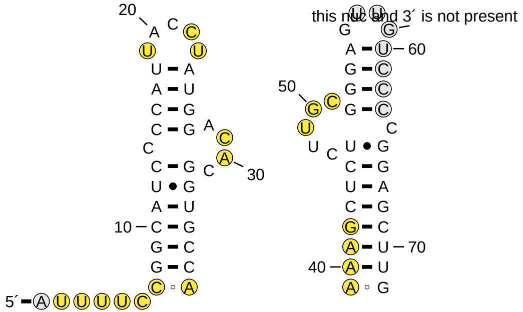

SAM-chlorobi NC_002932.3/706042-705555:X

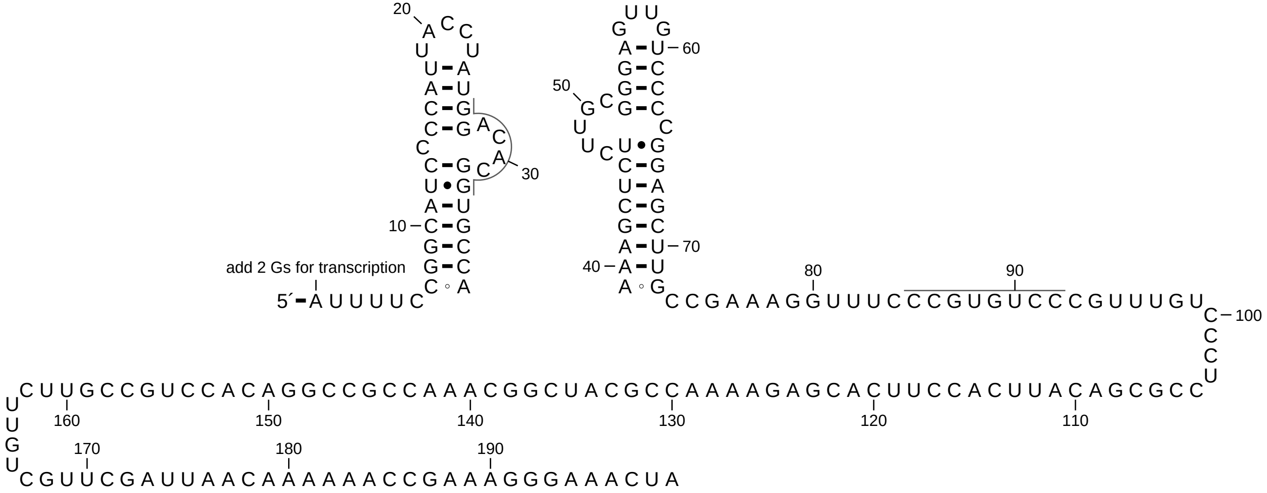

SAM-chlorobi-PKNOT

subfam_weight=1

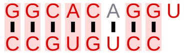

Supplement: Additional file 1 — Source code and example input and output files. C++ and Perl source code is provided. Installation and usage instructions are given in the user manual (Additional file 2), which is also a part of this archive. Example input files for R2R are included in the "demo" subdirectory of this archive, and R2R's raw output on these examples in PDF and SVG format is supplied in the "demo/output-pdf" and "demo/output-svg" subdirectories. Files can be retrieved from the tgz archive file using programs such as WinZip (Windows), StuffIt Expander (Mac) or the tar/gzip commands (UNIX). [file 1471-2105-12-3-S1.TGZ › R2R-1.0.1/demo/output-pdf/SAM-chlorobi.pdf]
